# Associations of dietary patterns and obesity development in school‐aged children: results from the CHILD Cohort Study

**DOI:** 10.1002/oby.24294

**Published:** 2025-06-18

**Authors:** Zheng Hao Chen, Gabrielle Jacobson, Myrtha E. Reyna, Paula Parvulescu, Russell J. de Souza, Mark R. Palmert, Wendy Lou, Susan C. Campisi, Elinor Simons, Stuart E. Turvey, Theo J. Moraes, Piushkumar J. Mandhane, Padmaja Subbarao, Kozeta Miliku

**Affiliations:** ^1^ Department of Nutritional Sciences University of Toronto Toronto Ontario Canada; ^2^ Department of Physiology University of Toronto Toronto Ontario Canada; ^3^ Department of Pediatrics, Hospital for Sick Children University of Toronto Toronto Ontario Canada; ^4^ Dalla Lana School of Public Health University of Toronto Toronto Ontario Canada; ^5^ Public Health Department Liverpool City Council Liverpool UK; ^6^ Department of Health Research Methods, Evidence, and Impact, Faculty of Health Sciences McMaster University Hamilton Ontario Canada; ^7^ Global Health Graduate Programs McMaster University Hamilton Ontario Canada; ^8^ Division of Endocrinology Hospital for Sick Children, University of Toronto Toronto Ontario Canada; ^9^ Department of Paediatrics and Child Health University of Manitoba Winnipeg Manitoba Canada; ^10^ Department of Pediatrics University of British Columbia Vancouver British Columbia Canada; ^11^ Department of Pediatrics University of Alberta Edmonton Alberta Canada; ^12^ Department of Medicine McMaster University Hamilton Ontario Canada

## Abstract

**Objective:**

We aimed to understand data‐driven dietary patterns in Canadian preschoolers and their impact on obesity development among male and female individuals.

**Methods:**

In the prospective, population‐based Canadian pregnancy cohort, the CHILD Cohort Study (*N* = 2219), dietary intake was assessed at age 3 years using a previously developed 112‐item food frequency questionnaire. At age 5 years, we measured height, weight, and waist circumference and calculated BMI and waist circumference *z* scores. Obesity was defined as BMI *z* score > 2. We used principal components analysis to derive dietary patterns and multivariable‐adjusted regression analyses to determine dietary patterns' associations with BMI and waist circumference *z* scores, as well as obesity status.

**Results:**

Among Canadian preschoolers, we identified three dietary patterns: “Prudent” (high in vegetables, fruits, legumes, and fish); “Western‐like” (high in fast foods, red/processed meats, and carbonated drinks); and “Refined Grain‐Snack” (high in refined grains, dairy, and salty snacks). At age 5 years, 4.7% of the children were living with obesity (3.1% male individuals and 1.6% female individuals). Females adhering to the Refined Grain‐Snack pattern had higher waist circumference *z* scores (β = 0.14; 95% CI: 0.03–0.25) and 2.74‐fold odds of living with obesity (95% CI: 1.29–5.85). No significant associations were observed among male individuals or with other dietary patterns and obesity outcomes among female individuals.

**Conclusions:**

Preschool dietary patterns are associated with sex‐biased obesity development, highlighting the need for further research to explore these differences and inform targeted obesity prevention strategies during this important developmental period.

**Study Importance**

**What is already known?**

- Diet is a major modifiable risk factor for noncommunicable disease development.
- Adult studies have suggested that unhealthy dietary patterns are associated with higher odds of developing obesity.

**What does this study add?**

- Among 3‐year‐old Canadian preschoolers, we identified three dietary patterns: “Prudent” (high in fruits, vegetables, legumes, eggs, and fish); “Western‐like” (high in fast foods, red/processed meats, and carbonated drinks); and “Refined Grain‐Snack” (high in refined grains, dairy, and salty snacks).
- Adherence to the Refined Grain‐Snack dietary pattern at age 3 years was associated with higher BMI and waist circumference and higher odds of living with obesity at age 5 years only among female individuals.

**How might these results change the direction of research or the focus of clinical practice?**

- These findings highlight the importance of considering sex‐biased responses to early childhood dietary patterns, which could shift research toward identifying biological and behavioral mechanisms underlying these differences. Clinically, they underscore the need for early, sex‐informed dietary recommendations or interventions, particularly for females.

## INTRODUCTION

The obesity epidemic is a growing concern worldwide, leading to a mortality rate of approximately 5 million people annually [1]. Obesity is a risk factor for numerous noncommunicable diseases, including type 2 diabetes, cardiovascular disease, and cancer [1]. Thirty percent of Canadian children are living with overweight or obesity, and these values are higher among males compared to females [2]. Understanding the predictive factors of obesity development in early life, primarily by assessing body mass index (BMI) and waist circumference, is important because childhood obesity tracks through life and increases the risk of long‐term health complications [3].

Diet is a well‐established modifiable risk factor for obesity [4]. Much of the published literature has examined the impact of individual macro‐ or micronutrients on obesity rather than dietary patterns, which provide a more comprehensive understanding of nutrient intakes and reflect habitual eating behaviors [5, 6]. These dietary patterns can be a priori (hypothesis‐driven, e.g., Dietary Approaches to Stop Hypertension [DASH]) [7] or a posteriori (data‐driven) [8]. Adult studies have consistently shown that high adherence to a “prudent” [9] and low adherence to a “Western” [10] or “modern” (similar characteristics of Western) [11] dietary pattern are associated with lower BMI [9, 10] and smaller waist circumference [11]. Interestingly, adult studies have also suggested sex‐biased associations between dietary patterns and obesity. For example, adherence to the prudent pattern in Taiwanese adults was associated with lower odds of obesity among male individuals [10].

Previous studies in children have examined dietary patterns [12]; however, there are limited studies that have examined data‐driven dietary patterns and their associations with obesity, particularly in Canadian preschoolers [13, 14, 15]. Cross‐sectional studies in school‐aged children have suggested potential benefits of healthy eating patterns, similar to those observed in adults. A New Zealand study found that a “healthy” dietary pattern (with similar characteristics to a prudent pattern) was associated with lower odds of obesity at age 6 years [13]. Additionally, a study in China found that 10‐year‐old children adhering to a Western dietary pattern had higher odds of abdominal obesity [14]. Despite the established link between dietary patterns and obesity in adults and school‐aged children, research in preschoolers remains scarce, yielding inconclusive findings. A cross‐sectional Australian study reported no significant association between dietary patterns and obesity risk among children aged 1 to 4 years [15]. Interestingly, a study in Portugal reported a positive association between adherence to the “energy‐dense foods” dietary pattern (with similar characteristics of a Western dietary pattern) at age 4 years and higher BMI values and obesity risk at age 10 years among female individuals [16]. These findings highlight the need for longitudinal research to understand the role of dietary patterns on obesity risk in early childhood, particularly potential sex‐biased associations.

Although some studies have suggested a relationship between dietary patterns and childhood obesity, the lack of sex‐stratified analysis and reliance on cross‐sectional designs leave gaps in our understanding. It is important to fully examine early childhood dietary patterns because they track into adulthood and may impact short‐ and long‐term health outcomes in a sex‐biased manner. In order to address this gap, we used data from the Canadian CHILD Cohort Study, a prospective pregnancy cohort, to determine the associations between preschool dietary patterns and later childhood outcomes such as BMI, waist circumference, and obesity at school‐age. We also examined sex as a potential modifier of these associations.

## METHODS

### Study population

The CHILD Cohort Study is a population‐based, prospective pregnancy cohort study with participants from four sites across Canada (Vancouver, Edmonton, Manitoba, and Toronto). Women with singleton pregnancies enrolled between 2008 and 2012 were eligible if they delivered a healthy infant at >35 weeks’ gestation with a normal birth weight and no congenital abnormalities [17]. This study was performed among children with dietary information at age 3 years (*N* = 2439). We excluded participants missing responses to 12 or more food items (*n* = 5) or those with daily energy intake outside three logged SD scores from the mean (*n* = 22) [18]. Finally, we restricted the analyses to participants with available data on BMI or waist circumference at age 5 years (*N* = 2219; Figure S1). This study was approved by the Human Research Ethics Boards at McMaster University; the Universities of Manitoba, Alberta, and British Columbia; and the Hospital for Sick Children.

### Assessment of dietary intake

Dietary intake was assessed at age 3 years using a semiquantitative food frequency questionnaire (FFQ) [19]. This FFQ was validated in a subset of the Family Atherosclerosis Monitoring in Early Life (FAMILY) Study using 3‐day food records among 3‐year‐old children for calcium and vitamin D intakes [20, 21]. The FFQ comprised 112 food and drink questions completed by the caregivers. A nine‐level scale from “none” to “>3/day” was used for quantifying the consumption of each food item, which was subsequently converted to daily intake [22]. Total daily energy intake was calculated using the 2012 Food and Nutrients Database for Dietary Studies from the US Department of Agriculture [23].

### Assessment of BMI, waist circumference, and obesity

At age 5 years, height was determined in a standing position to the nearest millimeter without shoes by a Harpenden stadiometer (Holtain), and weight was measured using a calibrated scale as previously reported [24]. Waist circumference (in centimeters) was obtained using a nonstretchable measuring tape (OHAUS Corp.) with an attached spring scale tension gauge. We calculated age‐ and sex‐adjusted BMI *z* scores using the World Health Organization (WHO) 2007 Child Growth Standards [25], which is recommended for Canadian health studies of participants aged 5 to 19 years [26]. Waist circumference *z* scores were calculated based on the Third National Health and Nutrition Examination Survey (NHANES III) LMS table [27]. We classified children living with obesity (BMI *z* score > 2) and overweight or obesity (BMI *z* score > 1) [25]. Additionally, we calculated BMI percentiles using the Centers for Disease Control and Prevention (CDC) 2000 age‐ and sex‐specific growth charts and defined obesity as BMI ≥ 95th percentile [28].

### Covariates

Maternal age, education level (completion of a postsecondary degree), household income, and perceived stress during pregnancy were collected via questionnaires at the enrollment visit [17, 29]. Maternal prepregnancy BMI was calculated from measured height and self‐reported prepregnancy weight, validated against health records in a subset of CHILD Cohort Study participants [30]. Child sex and gestational age and weight at birth were obtained from birth charts. Information on breastfeeding exclusivity in the first 3 months, breastfeeding duration, and introduction to solid foods was collected through repeated questionnaires. Information on child ethnicity (Caucasian White, multiracial, or other), having older siblings (yes vs. no), and screen time (hours per day) at age 5 years was assessed using questionnaires completed by caregivers. Information on the study site (Vancouver, Edmonton, Manitoba, or Toronto) and the season of FFQ data collection (spring, summer, winter, or autumn) was also obtained.

### Statistical analysis

To derive the dietary patterns at age 3 years, we grouped all of the FFQ food items into 28 food groups based on the Canadian Community Health Survey (CCHS) (Table S1) [31]. Then, we used principal components analysis (PCA), a commonly used tool in nutritional epidemiology, with varimax rotation to derive the dietary patterns [8, 32]. To determine the number of patterns/principal components, we used an eigenvalue cutoff of >1.5, supported by a visual evaluation of the scree plot. Food groups with a factor loading ≥ |0.3| were considered significant contributors to the pattern [32].

We used basic (adjusted for child total energy intake [kilocalories per day]) and multivariable‐adjusted linear (for BMI and waist circumference *z* scores) and logistic (for obesity and overweight/obesity) regression analyses to examine the associations of dietary patterns and study outcomes. The multivariable‐adjusted models accounted for maternal age, prepregnancy BMI, maternal education level, pregnancy stress, household income, study site, weight and gestational age at birth, child ethnicity, breastfeeding exclusivity and duration, having older siblings, total energy intake, and season of dietary assessments at age 3 years and screen time at age 5 years. These covariates were included in the models based on their associations with childhood diet and obesity outcomes in previous studies, following the confounder's rule and the model fit (*R* ^2^). Other covariates were tested but not included as they did not improve the model fit (e.g., time of introduction to solid foods).

To assess whether associations differed by child sex, we evaluated the statistical interaction by including the product term with dietary patterns in the models. Significant interaction (*p* < 0.05) was observed for child sex; therefore, we conducted stratified analyses by sex. In order to account for reverse causality, in a sensitivity analysis, we restricted our study population to all individuals with BMI *z* score data at age 1 year and accounted for BMI *z* scores at age 1 year in the final model (*n* = 2162). In another sensitivity analysis, we additionally adjusted in final models for the change in energy intake between age 3 and 5 years and BMI *z* score data at age 3 years (*n* = 1898).

To reduce potential bias associated with missing covariate data, missing values of covariates (household income, maternal factors [age, prepregnancy BMI, education, and stress levels], birth weight, gestational age, child ethnicity, breastfeeding duration, exclusive breastfeeding at age 3 months, and screen time) were imputed (*n* = 10 imputations) according to the fully conditional specification method (predictive mean matching) and assuming no monotone missing pattern [33]. We report the pooled effect estimates after the multiple imputation procedure [34]. Participant characteristics before and after imputation are given in Table S2. *P* values < 0.017 (0.05/3 dietary patterns) were considered statistically significant after applying false discovery rate correction for multiple testing using the Bonferroni correction method [35].

Analyses were performed using SAS version 9.4 (Enterprise Guide 6.1, SAS Institute Inc.) and SPSS Statistics version 29.0 (IBM Corp.). Figures were generated using the “ggplot2” packages in RStudio version 2024.04.2 (Posit PBC).

## RESULTS

Table 1 shows study participant characteristics. Diet was assessed at the median age of 3.0 years (IQR 3.0, 3.1), and median energy intake at age 3 years was 1395 kcal (IQR 1142, 1706). The median age at outcome measurement was 5.0 years (IQR 5.0, 5.1). At the 5‐year visit, 4.7% of the study participants were classified as living with obesity (3.1% male individuals and 1.6% female individuals), and 20.0% of participants were classified as living with overweight or obesity (11.8% male individuals and 8.2% female individuals). These characteristics were similar among participants in the full cohort (Table S3).

**TABLE 1:** Participant characteristics.

	All children included in the analyses
	(*N* = 2219)
Family characteristics^a^	
Maternal age, y	32.5 (4.5)
Maternal BMI, kg/m^2^	23.1 [21.0, 26.7]
Maternal education (postsecondary education vs. none)	1715 (79.1)
Household income	
$0–$99,999	914 (42.2)
≥$100,000	1061 (47.8)
Prefer not say/skipped	192 (8.7)
Pregnancy stress score	11.1 [8.0, 16.0]
Study site	
Edmonton	492 (22.2)
Manitoba	705 (31.8)
Toronto	483 (21.7)
Vancouver	539 (24.3)
Birth characteristics^a^	
Child sex (male vs. female)	1175 (53.0)
Birth weight, g	3457.9 (476.1)
Gestational age at birth, wk	39.7 [38.9, 40.6]
Child ethnicity^b^	
Caucasian White	1447 (65.2)
Multiracial	512 (23.1)
Other	252 (11.3)
Older siblings (yes vs. no)	995 (44.8)
Exclusive breastfeeding at age 3 mo, (yes vs. no)	1371 (61.8)
Breastfeeding duration, mo	11.0 [6.0, 15.0]
Childhood characteristics	
Age at dietary assessment, y	3.0 [3.0, 3.1]
Daily caloric intake at age 3 y, kcal/d	1395.1 [1142.0, 1706.0]
Study season at 3‐y diet assessment	
Autumn	518 (23.3)
Spring	603 (27.2)
Summer	563 (25.4)
Winter	535 (24.1)
Age at outcome assessment, y	5.0 [5.0, 5.1]
TV watching at age 5 y, h/d	3.0 [2.0, 4.5]
Body composition and obesity	
BMI *z* score (WHO) at age 5 y	0.3 (1.0)
Male	0.4 (1.0)
Female	0.3 (0.9)
Waist circumference *z* score (NHANES III) at age 5 y	−0.1 (1.0)
Male	−0.1 (1.0)
Female	−0.1 (0.9)
Obesity + overweight at age 5 y (yes vs. no)	444 (20.0)
Male	261 (11.8)
Female	183 (8.2)
Obesity at age 5 y (yes vs. no)	105 (4.7)
Male	69 (3.1)
Female	36 (1.6)

Abbreviations: NHANES III, The Third National Health and Nutrition Examination Survey; WHO, World Health Organization.

^a^ Values are frequency counts and percentages for categorical variables, mean (SD) for continuous variables with a normal distribution, or median [IQR; 25th, 75th percentiles] for continuous variables with a skewed distribution. Values are based on the original data.

^b^ If both parents' ethnicities are Caucasian White, then the child's ethnicity is Caucasian White. If both parents' ethnicities are different from one another (and not “other”) or both ethnicities are multiracial, the child is defined as multiracial. If either parent's ethnicity is “other” (e.g., the following provided options did not apply: Black, Caucasian White, East Asian, First Nations, Hispanic, Middle Eastern, multiracial, South Asian, and Southeast Asian), then the child is defined as “other” as well.

### Dietary patterns characteristics

We identified three PCA‐derived dietary patterns: a “Prudent” dietary pattern characterized by high loadings of vegetables, fruits, legumes, fish, eggs, and poultry; a “Western‐like” dietary pattern characterized by high loadings of fast foods, red/processed meats, fruit juice, carbonated drinks, desserts, snacks, and pasta/rice; and a novel dietary pattern, which we called “Refined Grain‐Snack,” characterized by high loadings of refined grains, dairy, salty snacks, and nuts and a low intake of milk substitute. Together, these three PCA‐derived patterns explained ~30% of the total variance in food intake, which is consistent with previous literature (Table 2) [32].

**TABLE 2:** Dietary patterns from principal components analysis at age 3 years in the CHILD Cohort Study.

Food groups	Prudent	Western‐like	Refined Grain‐Snack
Other vegetables	**0.8**	0.0	0.0
Green vegetables	**0.7**	−0.1	0.0
Orange vegetables	**0.7**	−0.1	**0.3**
Legumes	**0.6**	−0.1	−0.1
Fruits	**0.5**	0.1	**0.3**
Starchy vegetables	**0.5**	0.2	0.1
Fish	**0.5**	0.2	−0.2
Eggs	**0.4**	0.1	0.0
Poultry	**0.4**	0.2	−0.1
Whole grains	0.2	−0.1	0.1
Fast foods	0.0	**0.7**	0.1
Sweet snacks	0.0	**0.6**	0.1
Meats	0.2	**0.6**	−0.1
Processed meats	0.1	**0.6**	0.1
Fruit juice	0.0	**0.5**	0.0
Cakes/cookies	0.0	**0.5**	**0.3**
Carbonated drinks	0.0	**0.4**	−0.1
Pancakes	−0.1	**0.3**	0.1
Pasta/rice	0.1	**0.3**	0.1
Refined grains	0.0	0.2	**0.6**
Cheese	0.1	0.1	**0.5**
Solid fat	0.0	0.2	**0.5**
Milk substitutes	**0.3**	0.2	**−0.4**
Yogurt	0.1	0.2	**0.4**
Nuts	0.2	−0.1	**0.4**
Salty snacks	0.0	**0.3**	**0.4**
Milk	−0.1	−0.1	**0.3**
Sauce dressings	0.2	0.2	**0.3**

*Note*: Dietary patterns (Prudent, Western‐like, and Refined Grain‐Snack) include food goups that significantly contributed to that pattern (factor loading ≥ |0.3|) Bold values denote significant contributors. Darker shading indicates the food group contributes more strongly to the dietary pattern.

### Associations of dietary patterns at age 3 years with BMI and waist circumference *z* scores at age 5 years

**FIGURE 1:**
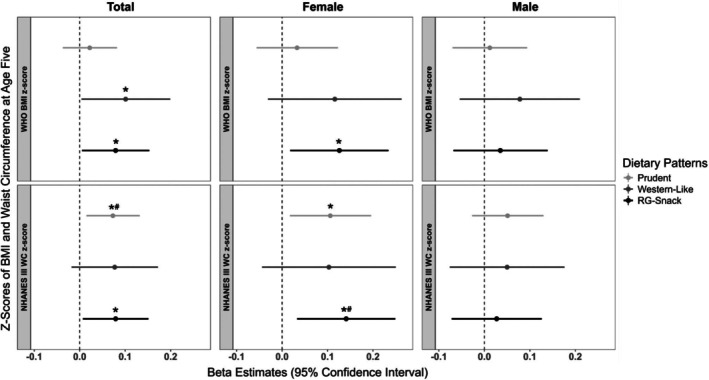
Associations of dietary patterns at age 3 years and BMI and waist circumference *z* scores at age 5 years, stratified by sex in the CHILD Cohort Study (*N* = 2219). Values are β estimates with 95% CI from linear regression analyses of dietary patterns at age 3 years (Prudent, Western‐like, and Refined Grain‐Snack [RG‐Snack]) and BMI and waist circumference *z* scores at age 5 years in the CHILD Cohort Study (*N* = 2219), stratified by female (*n* = 1044) and male (*n* = 1175) individuals. The multivariable‐adjusted analyses accounted for maternal age, prepregnancy BMI, postsecondary education, pregnancy stress, household income, study site, weight and gestational age at birth, child ethnicity, breastfeeding exclusivity and duration, having older siblings, screen time, total energy intake at age 3 years, and season of dietary assessments. *p < 0.05, and “#” indicates that significance remained after Bonferroni correction. NHANES III, The Third National Health and Nutrition Examination Survey; WHO, World Health Organization.

Figure 1 shows the associations of child dietary patterns at age 3 years with BMI and waist circumference *z* scores at age 5 years. We observed consistent associations of our novel Refined Grain‐Snack dietary pattern (characterized by a high intake of refined grains, dairy, nuts, and salty snacks) with BMI and waist circumference *z* scores. In the multivariable‐adjusted models, among all children, high adherence to the Refined Grain‐Snack dietary pattern was associated with 0.08‐higher BMI *z* scores (95% confidence interval [CI]: 0.01–0.15) (Figure 1). This association was primarily driven by female individuals (β = 0.13, 95% CI: 0.02–0.23). However, after accounting for multiple testing, none of these associations retained statistical significance. No significant associations were observed among male individuals in the multivariable‐adjusted model. The results from the basic models were shown in Figure S2 and reflect similar but stronger effect estimates between the Refined Grain‐Snack dietary pattern and BMI *z* scores.

Among all children, the Refined Grain‐Snack dietary pattern was also associated with larger waist circumference *z* scores at age 5 years (Figure 1 and Figure S2). Similar to the BMI findings, we observed sex‐biased associations between the Refined Grain‐Snack dietary pattern with waist circumference *z* scores. Among female individuals, in the multivariable‐adjusted analysis, adherence to the Refined Grain‐Snack pattern was associated with 0.14‐higher (95% CI: 0.03 to 0.25) waist circumference *z* scores (Figure 1). This association remained significant after accounting for multiple testing. The associations between the Refined Grain‐Snack dietary pattern and waist circumference *z* scores remained significant when additionally accounting for BMI *z* scores at age 1 year (Figure S3) and when accounting for BMI *z* scores at age 3 years and the change in energy intake from age 3 to 5 years (Figure S4). The association between the Refined Grain‐Snack pattern and waist circumference was not statistically significant among male individuals (β = 0.03; 95% CI: −0.07 to 0.13; Figure 1).

We also observed associations between the Prudent dietary pattern (characterized by a high intake of vegetables, fruits, legumes, fish, eggs, and poultry) and waist circumference *z* scores, but not BMI *z* scores. Among all children, in the multivariable‐adjusted linear regression analyses, adherence to the Prudent dietary pattern was associated with 0.07‐larger (95% CI: 0.02–0.13) waist circumference *z* scores at age 5 years (Figure 1). This association also appeared to be sex‐biased toward female individuals (β = 0.14; 95% CI: 0.02–0.20); however, among female individuals, it did not pass the significance threshold after accounting for multiple testing (Figure 1).

Lastly, in the multivariable‐adjusted models, among all children, adherence to the Western‐like dietary pattern (characterized by high intake of fast foods, red/processed meats, fruit juice, carbonated drinks, desserts, snacks, and pasta/rice) was associated with higher BMI *z* scores (β = 0.10, 95% CI: 0.004–0.20). This association did not retain significance after multiple testing adjustments (Figure 1). No significant associations were observed between the Western‐like dietary pattern and waist circumference *z* scores. Not all of the associations observed in the basic model (e.g., Western‐like dietary pattern and BMI *z* scores; Figure S2) retained significance when accounting for the study covariates.

### Associations of dietary patterns at age 3 years with obesity status at age 5 years

**FIGURE 2:**
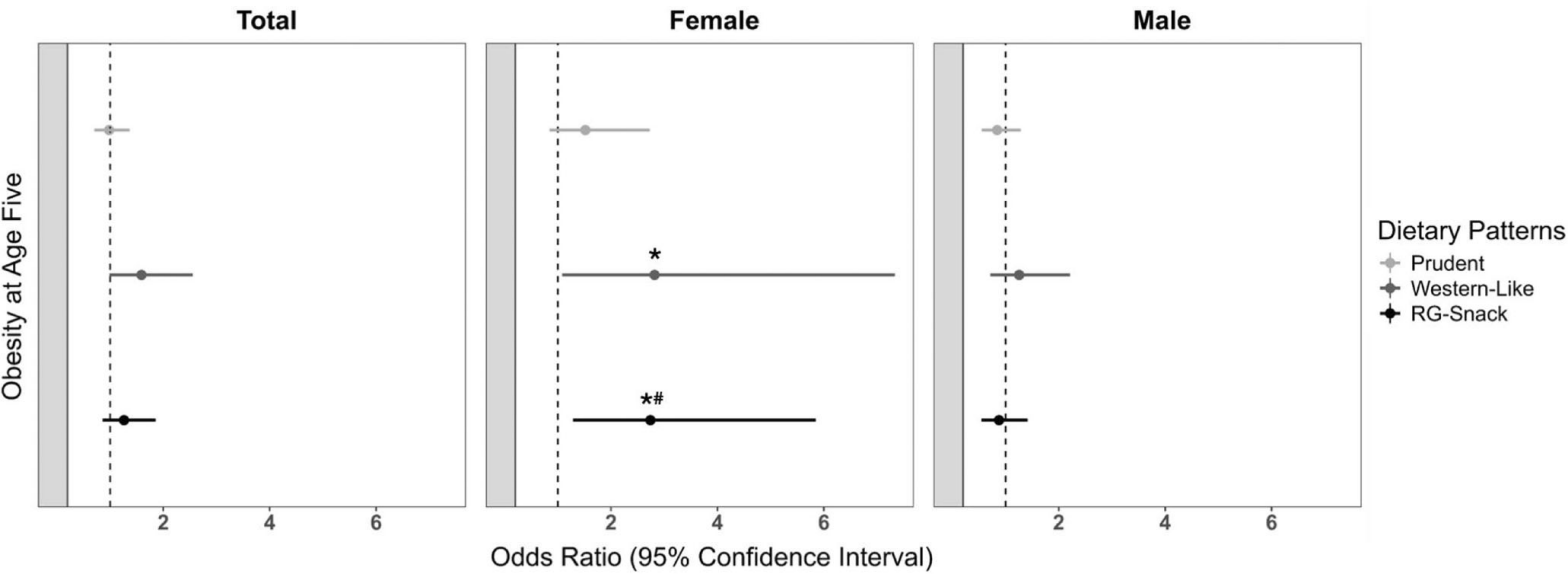
Associations of dietary patterns at age 3 years and obesity status at age 5 years, stratified by sex in the CHILD Cohort Study (*N* = 2216). Values are odds ratios with 95% CI from logistic regression analyses of dietary patterns at age 3 years (Prudent, Western‐like, and Refined Grain‐Snack [RG‐Snack]) and obesity status at age 5 years (WHO BMI *z* score > 2) in the CHILD Cohort Study (*N* = 2216), stratified by female (*n* = 1044) and male (*n* = 1172) individuals. The multivariable‐adjusted models accounted for maternal age, prepregnancy BMI, postsecondary education, pregnancy stress, household income, study site, weight and gestational age at birth, child ethnicity, breastfeeding exclusivity and duration, presence of older siblings, screen time, total energy intake at age 3 years, and season of dietary assessments. *p < 0.05, and “#” indicates significance remained after Bonferroni correction.

At the 5‐year visit, 105 (4.7%) of the study participants were living with obesity: 69 (3.1%) were male, and 36 (1.6%) were female (Table 1). In the multivariable‐adjusted logistic regression analyses, high adherence to the Refined Grain‐Snack dietary pattern at age 3 years was associated with 2.74‐higher (95% CI: 1.29–5.85) odds of living with obesity at age 5 years among female individuals after accounting for multiple testing (Figure 2). These results were consistent with slightly smaller effect estimates when obesity was defined based on the CDC percentiles (odds ratio [OR] 2.56, 95% CI: 1.42–4.64; Figure S5). No statistically significant associations were observed among male individuals (OR 0.88, 95% CI: 0.55–1.41) (Figure 2). Furthermore, also among female individuals, a higher adherence to the Refined Grain‐Snack dietary pattern was significantly associated with 1.88‐higher (95% CI: 1.29–2.74) odds of living with overweight or obesity (Figure S6). The associations of the Western‐like dietary pattern and obesity did not retain statistical significance in the multivariable‐adjusted models and after accounting for multiple testing (Figures 2 and S5).

## DISCUSSION

Our study identified three dietary patterns among healthy Canadian preschoolers at 3 years of age: a “Prudent” pattern (high loadings of vegetables, fruits, legumes, fish, eggs, and poultry), a “Western‐like” pattern (high loadings of fast foods, red/processed meats, fruit juice, carbonated drinks, desserts, snacks, and pasta/rice), and a “Refined Grain‐Snack” pattern (high loadings of refined grains, dairy, salty snacks, and nuts). We found a novel association between higher adherence to the Refined Grain‐Snack dietary pattern at age 3 years with higher waist circumference and higher odds of living with obesity at age 5 years. Finally, we also found that the associations between dietary patterns and obesity development were primarily biased toward female individuals.

Nutritional epidemiology has undergone a paradigm shift in recent decades, moving from a focus on individual nutrients to a more holistic approach that examines dietary patterns. The field now prioritizes the investigation of dietary patterns, which can be either hypothesis‐driven, informed by existing knowledge, or data‐driven, discovered directly from dietary intake analysis. PCA (data‐driven) is a common tool used for the identification of dietary patterns [8]. Although the nomenclature of the dietary patterns is subjective, terminology consistent with existing literature is used in this study [36]. Our Prudent dietary pattern aligns with other “health‐conscious” or “Mediterranean” patterns, characterized by high intakes of fruits and vegetables, along with fish, seafood, legumes, and whole grains [37, 38]. Similarly, our Western‐like pattern resembles established “unhealthy” or “Western” patterns rich in high‐fat foods, meats, fast foods, snacks, desserts and cookies, and sugary drinks [37, 38]. Notably, the Refined Grain‐Snack pattern, specifically high in refined grains, dairy, and snacks, appears to be unique and to our knowledge not reported in other studies focused on the preschool age group.

Studies exploring associations of preschool data‐driven dietary patterns with obesity have been inconsistent [13, 14, 15, 37]. These inconsistencies might be due to different definitions of dietary patterns and obesity‐related outcomes, different geographic locations, and the age of the children; and most studies have not examined the differing associations in male and female individuals separately. In addition, most studies on preschool diets are cross‐sectional. Among the limited prospective studies, one birth cohort in the Netherlands found that a health‐conscious dietary pattern (high loadings of vegetables, legumes, and fruits) at age 1 year, which shares similar characteristics with our Prudent dietary pattern, was not significantly associated with BMI at age 6 years [37]. This finding is consistent with our results. Contrary to findings in adult studies, surprisingly, we found that adherence to the Prudent dietary pattern was associated with higher waist circumference at age 5 years [39, 40]. Future studies are needed to validate our findings.

Previous studies have also reported that patterns with similar characteristics to our Western‐like pattern are associated with obesity‐related outcomes. For example, among Portuguese children, adherence to the “energy‐dense foods” dietary pattern at age 4 years, which has similarities to our Western‐like pattern, was associated with increased BMI among female individuals only at age 10 years [16]. In our study, adherence to the Western‐like pattern was associated with higher BMI *z* scores in female individuals in the basic models; however, it did not retain statistical significance after accounting for maternal BMI, birth factors, and child screen time. Interestingly, our new Refined Grain‐Snack pattern was associated with waist circumference and obesity among female individuals. These results remained consistent even when accounting for baseline BMI and the change in energy intake from age 3 to 5 years. A meta‐analysis of observational studies in children found that a higher intake of refined grains was associated with increased odds of overweight/obesity; however, comparison with our study is limited as the study did not explore sex‐biased associations [41]. The energy‐dense food items in the Refined Grain‐Snack dietary pattern may drive the sex‐biased associations, as a previous study found that fruit‐based yogurt was significantly associated with body fat percentage among Polish school‐aged female individuals [42].

Research has consistently shown global sex differences in childhood obesity development, with a higher prevalence observed in male compared to female individuals [2]. While, after birth, female individuals generally have greater fat mass and subcutaneous adipose tissue than male individuals, female individuals also have higher circulating concentrations of leptin, an appetite‐suppressant hormone [2]. Estrogen levels may also account for differences in energy homeostasis or the development of insulin insensitivity [43]. This difference in both leptin and insulin sensitivity between male and female individuals leads to the hypothesis that female individuals are physiologically more inclined to store and use fat than protein as, from an evolutionary standpoint, it is an adaptive response to reproductive success [43]. Meanwhile, male individuals rely more on glucose and protein metabolism [43].

Although our study effect sizes may be small, they may be significant on a population level and can inform clinical practice and public health guidelines. Through this research and further work, dietary recommendations could be modified during critical periods of development in early childhood. Owing to the sexual dimorphism already seen at this age in response to dietary choices, this research can inform health care professionals on personalized dietary recommendations to reduce obesity among children. Our work forms the epidemiological foundation for understanding sex‐biased differences in how dietary patterns influence growth and obesity risk during early childhood. However, future studies are needed to validate our findings and expand their implications in policy and health care settings. While we observed significant associations among female individuals, we acknowledge that specifically targeting dietary recommendations toward female preschoolers, without providing them with adequate nutritional context, may have unintended consequences, such as developing disordered eating behaviors and nutritional deficiencies. This work can inform inclusive (pre)school food programs and public health interventions on promoting balanced diets through positive messaging that emphasizes variety, enjoyment, and nutritional value of healthy food choices and guides both male and female preschoolers on limiting refined grains as a large part of their diet.

To our knowledge, our study is Canada's first and largest population‐based prospective cohort study examining dietary patterns in preschool children. Both our exposure and outcomes were collected using standardized tools. Obesity was defined using WHO cutoffs, which are recommended for Canadian children's growth when compared to other international cutoff systems [44, 45]. To increase the generalizability of our findings, we also defined obesity using BMI percentiles based on CDC cutoffs and observed similar results. The large and detailed CHILD Cohort Study database allowed us to control our models for various lifestyle, health, and environmental factors, some of which had been missed in previous studies. We selected the confounders a priori, based on the confounder's rule and model fit. Furthermore, the large study size gave us the opportunity to run the analyses among male and female individuals, which addresses a research gap in the literature.

Our study has some limitations. For example, the FFQ data have inherent limitations, such as recall bias or limited food lists. Although our FFQ has been validated in a subset of the FAMILY Study [20, 21], this nutrition tool has limitations, as it was validated against another self‐reported tool. In addition, a proportion of our participants was lost to follow‐up or missed diet data at age 3 years and obesity‐related data at age 5 years. However, when we compared our study participants' characteristics with all CHILD Cohort Study participants, they were similar. Finally, although we adjusted for many potential confounders, residual confounding for the observed associations might be present.

## CONCLUSION

In this large, multiethnic, population‐based prospective cohort study, we identified three novel dietary patterns in Canadian preschoolers and showed that the “Refined Grain‐Snack” dietary pattern was associated with higher waist circumference and odds of obesity, primarily among female individuals. These findings are important, as dietary patterns are established in early childhood and childhood obesity tracks in adulthood. Further research in this age group is needed to confirm our findings and to understand the sex‐related differences in the association of diet with obesity development.

## AUTHOR CONTRIBUTIONS

Kozeta Miliku and Padmaja Subbarao designed this project. Kozeta Miliku managed the project. The CHILD Study Founding team (Wendy Lou, Elinor Simons, Stuart E. Turvey, Theo J. Moraes, Piushkumar J. Mandhane, and Padmaja Subbarao) conceived the CHILD cohort design, managed study recruitment and oversaw clinical assessments of study participants. Zheng Hao Chen, Myrtha E. Reyna, Paula Parvulescu, Russell J. de Souza, and Kozeta Miliku compiled the nutrient matrix from which energy and macro‐ and micronutrient data were derived. Zheng Hao Chen and Gabrielle Jacobson conducted all the statistical analyses. Myrtha E. Reyna and Kozeta Miliku oversaw the data analyses. Mark R. Palmert provided support with the clinical interpretation of the data. Susan C. Campisi advised on the food groups. Zheng Hao Chen, Gabrielle Jacobson, and Kozeta Miliku interpreted the data and drafted the manuscript. All authors provided feedback and approved the final version. Zheng Hao Chen and Gabrielle Jacobson have full access to the data in the study and take responsibility for the integrity of the data and the accuracy of the data analysis.

## FUNDING INFORMATION

This research was funded by the Canadian Institutes of Health Research (CIHR) and the Allergy, Genes and Environment (AllerGen) Network of Centres of Excellence (NCE), and Genome Canada provided core funding for CHILD Cohort Study. These entities had no role in the design and conduct of the study; collection, management, analysis, and interpretation of the data; preparation, review, or approval of the manuscript; or decision to submit the manuscript for publication.

## CONFLICT OF INTEREST STATEMENT

The authors declared no conflicts of interest.

## Supporting information


**Data S1.** Supporting Information.

## Data Availability

Data are available upon request. Researchers interested in developing or collaborating on a project using CHILD data are encouraged to contact the study's National Coordinating Centre for a formal request. Details for data request and access are outlined on the CHILD website: https://childstudy.ca/for-researchers/data-access/
