# Supplementary material for: Associations of dietary patterns and obesity development in school‐aged children: results from the CHILD Cohort Study
Source: Obesity (Silver Spring). 2025 Jun 18;33(7):1355–64. doi: 10.1002/oby.24294 (PMC12210095; doi:10.1002/oby.24294)
Supplement: Supplementary file 1 — Data S1. Supporting Information. [file OBY-33-1355-s001.docx]

Supplementary Materials

**Associations of dietary patterns and obesity development in school-aged children: Results from the CHILD Cohort Study**

Zheng Hao Chen† ^1^,Gabrielle Jacobson† ^2,3^, Myrtha E. Reyna ^3, 4^, Paula Parvulescu ^5^,
Russell J. de Souza ^6,7^, Mark R. Palmert ^2,3,8^, Wendy Lou ^4^, Susan C Campisi ^4^, Elinor Simons ^9^, Stuart E. Turvey ^10^, Theo J. Moraes ^2^, Piushkumar J. Mandhane ^11^, Padmaja Subbarao* ^2,4,12^, Kozeta Miliku* ^1,12^

| **Figure S1** | Flowchart of study participants included for the analysis |
| --- | --- |
| **Table S1** | Mapping of food frequency questionnaire items based on CCHS food groups |
| **Table S2** | Descriptive characteristics in the original and imputed dataset |
| **Table S3** | Descriptive characteristics of participants in the full cohort vs those included in the analysis: the CHILD Cohort Study |
| **Figure S2** | Associations of dietary patterns at three years of age and body mass index and waist circumference z-scores at five years of age from the basic model, stratified by sex in the CHILD Cohort Study (N=2219) |
| **Figure S3** | Multivariable-adjusted associations of dietary patterns, body mass index and waist-circumference z-scores additionally accounting for one-year body mass index, stratified by sex in the CHILD Cohort Study (N=2162) |
| **Figure S4** | Multivariable-adjusted associations of dietary patterns, body mass index and waist-circumference z-scores additionally accounting for three-year body mass index and change in energy intake, stratified by sex in the CHILD Cohort Study (N=1898) |
| **Figure S5** | Multivariable-adjusted associations of dietary patterns at three years of age and obesity status (CDC Standards) at five years of age, stratified by sex in the CHILD Cohort Study (N=2217) |
| **Figure S6** | Multivariable-adjusted associations of dietary patterns at three years of age and overweight/obesity status at five years of age, stratified by sex in the CHILD Cohort Study (N=2216) |
|  |  |
|  |  |

## **Figure S1**. Flowchart of study participants included for the analysis

## **Table S1.** Mapping of food frequency questionnaire items based on CCHS food groups

Participants eligible for the three-year visit

**N = 3232**

Participants with FFQ data available at age three

**N = 2412**

Participants with no FFQ data available at age three (N=793), <12 questions missing (~10%) from FFQ (N=5) or outliers calculated FFQ energy intake (N=22)

**N = 820**

Participants eligible at birth

**N = 3454**

Participants lost to follow-up

**N = 222**

Participants with no data available for body mass index, or waist circumference z-scores

**N = 193**

Pregnant women recruited in the CHILD study

**N = 3621**

Participants not meeting the eligibility criteria

**N = 167**

Participants with FFQ data at age three and one or both clinical measures at age five

**N = 2219**

With data at 5 available on:

Body mass index **N = 2216**

Waist circumference **N = 2201**

| **CCHS Food Groups** | **CHILD Food Items** |
| --- | --- |
| **Fast Foods** | Pizza |
|  | French Fries, Fried Potatoes, Tater Tots |
|  | Hamburger |
|  | Weiner |
|  | Tacos, Burritos |
|  | Fried Chicken, Chicken Nuggets |
| **Pasta/ Rice** | Rice (Fried, Cooked) |
|  | Macaroni & Cheese |
|  | Spaghetti Or Other Pasta |
|  | Lasagna |
| **Refined Grains** | Bread-Pita |
|  | Bread- Toast |
|  | Corn Bread Or Tortilla |
|  | French Toast |
|  | Cold Cereal |
| **Whole Grains** | Hot Cereal, Grits |
| **Pancakes And Waffles** | Pancakes |
|  | Waffles |
| **Cakes/ Cookies** | Pie |
|  | Doughnut |
|  | Cookies Or Brownies |
|  | Cake Or Cupcake |
|  | English Muffin |
|  | Bagel |
|  | Egg McMuffin |
|  | Sweet Roll Or Muffin |
|  | Tea Biscuit |
| **Starchy Vegetables** | Sweet Potatoes Or Yams (Cooked) |
|  | Potatoes (Baked, Boiled Or Mashed) |
|  | Peas (Cooked) |
|  | Corn (Cooked) |
| **Orange Vegetables** | Carrot |
|  | Squash, Orange Or Winter |
|  | Tomatoes |
| **(Dark) Green Vegetables** | Broccoli |
|  | Spinach |
|  | Greens (Mustard, Turnip, Kale) |
|  | Lettuce Salad |
| **Other Vegetables** | Peppers |
|  | Green Beans |
|  | Cabbage, Coleslaw |
|  | Cauliflower |
|  | Zucchini, Yellow Squash |
|  | Mixed Vegetables |
|  | Vegetable Soup |
|  | Other Soup |
| **Legumes And Soy** | Beans |
|  | Lentils |
|  | Tofu |
| **Whole Fruits** | Banana |
|  | Peaches |
|  | Fruit Cocktail, Mixed Fruit |
|  | Orange |
|  | Grapefruit |
|  | Apple |
|  | Pear |
|  | Grapes |
|  | Strawberries |
|  | Watermelon |
|  | Cantaloupe |
|  | Pineapple |
|  | Applesauce |
|  | Dried Fruit |
| **Fruit Juice** | Orange Juice, Grapefruit Juice |
|  | Other Juice (Apples, Mango, Nectarine) |
| **Whole-Fat Milk** | Cow Milk |
|  | Goat Milk |
| **Cheese** | Cheese (Regular) |
|  | Cheese (Low Fat) |
|  | Cheese Spread (Cheese Whiz) |
|  | Cream Cheese |
| **Yogurt** | Yogurt |
|  | Mini Go |
|  | Yogurt Drink |
| **Eggs** | Eggs |
| **Fish And Shellfish** | Shrimp |
|  | Canned Tuna |
|  | Other Fish |
|  | Fried Fish, Fish Sticks |
| **Nuts, Seeds and Nut Butters** | Nuts |
|  | Peanut Butter |
| **Milk Substitutes** | Rice Milk |
|  | Almond Milk |
|  | Soy Drink |
| **Meat (Beef, Game and Organ Meats Veal, Lamb and Pork)** | Pork Or Ham |
|  | Roast Beef Or Steak |
|  | Meatballs |
|  | Liver, Organ Meats |
| **Poultry** | Stir Fried Chicken |
|  | Other Chicken Or Turkey |
| **Processed Meat** | Luncheon Meat |
|  | Bacon |
|  | Cold Cuts |
|  | Sausage |
| **Sweet Snacks** | Chocolate Or Candy Bar |
|  | Other Candy, Not Chocolate |
|  | Jello |
|  | Hot Chocolate |
|  | Ice Cream |
|  | Sorbet |
|  | Puddings |
|  | Granola Bar |
|  | Milk Shake |
| **Salty Snacks** | Potatoes Chips |
|  | Corn Chips (Nachos) |
|  | Crackers |
|  | Popcorn Or Pretzels |
| **Carbonated Drinks** | Soda, Soft Drinks, Pop (Regular) |
|  | Soda, Soft Drinks, Pop (Sugar Free) |
| **Solid Fat** | Margarine |
|  | Butter |
| **Sauces and Dressings** | Salad Dressing |
|  | Mayonnaise |

CCHS=Canadian Community Health Survey

## **Table S2**. Descriptive characteristics in the original and imputed dataset

|  | **Non-Imputed Data** | **Imputed Data** |
| --- | --- | --- |
|  |  |  |
|  | **(N = 2219)** | **(N = 2219)** |
| **Family Characteristics** |  |  |
| Maternal Age (years) | 32.5 (4.5) | 32.5 (4.5) |
| Missing (n, %) | 0 (0.0) |  |
| Maternal BMI (kg/m^2^) | 23.1 [21.0, 26.7] | 23.1 [21.0, 26.7] |
| Missing (n, %) | 67 (3.0) |  |
| Maternal Education (Post-secondary Education vs. None) | 1715 (77.3) | 1748 (78.8) |
| Missing (n, %) | 52 (2.3) |  |
| Annual Family Income |  |  |
| $0 - $99,999 | 914 (41.2) | 949 (42.8) |
| > $100,000 | 1061 (47.8) | 1075 (48.4) |
| Prefer Not to Say | 192 (8.7) | 195 (8.8) |
| Missing (n, %) | 52 (2.3) |  |
| Pregnancy Stress Score | 11.1 [8.0, 16.0] | 11.4 [8.0, 16.0] |
| Missing (n, %) | 132 (5.9) |  |
| Study Site |  |  |
| Edmonton | 492 (22.2) | 492 (22.2) |
| Manitoba | 705 (31.8) | 705 (31.8) |
| Toronto | 483 (21.7) | 483 (21.7) |
| Vancouver | 539 (24.3) | 539 (24.3) |
| Missing (n, %) | 0 (0.0) |  |
| **Birth Characteristics** |  |  |
| Child Sex (Males vs. Females) | 1175 (53.0) | 1175 (53.0) |
| Missing (n, %) | 0 (0.0) |  |
| Birth Weight (grams) | 3457.9 (476.1) | 3459.17 (476.2) |
| Missing (n, %) | 45 (2.0) |  |
| Gestational Age at Birth (weeks) | 39.7 [38.9, 40.6] | 39.7 [38.9, 40.6] |
| Missing (n, %) | 32 (1.4) |  |
| C-section Birth (Yes vs. No) | 543 (24.5) | 552 (24.9) |
| Missing (n, %) | 33 (1.5) |  |
| Child Ethnicity |  |  |
| Caucasian White | 1447 (65.2) | 1450 (65.4) |
| Multiracial | 512 (23.1) | 515 (23.2) |
| Other | 252 (11.3) | 254 (11.4) |
| Missing (n, %) | 8 (0.4) |  |
| Older Siblings (Yes vs. No) | 995 (44.8) | 1009 (45.5) |
| Missing (n, %) | 28 (1.3) |  |
| Exclusive Breastfeeding at 3 Months (Yes vs. No) | 1371 (61.8) | 1376 (62.0) |
| Missing (n, %) | 7 (0.3) |  |
| Breastfeeding Duration (months) | 11.0 [6.0, 15.0] | 11.0 [6.0, 15.0] |
| Missing (n, %) | 5 (0.2) |  |
| **Childhood Characteristics** |  |  |
| Exact Assessment Age at 3 Years Visit (years) | 3.0 [3.0, 3.1] | 3.0 [3.0, 3.1] |
| Missing (n, %) | 45 (2.0) |  |
| Daily Caloric Intake at 3 Years (kcal/day) | 1395.1 [1142.0, 1706.0] | 1395.1 [1142.0, 1706.0] |
| Missing (n, %) | 0 (0.0) |  |
| Study Season at 3 Years Diet Assessment |  |  |
| Autumn | 518 (23.3) | 518 (23.3) |
| Spring | 603 (27.2) | 603 (27.2) |
| Summer | 563 (25.4) | 563 (25.4) |
| Winter | 535 (24.1) | 535 (24.1) |
| Missing (n, %) | 0 (0.0) |  |
| Exact Assessment Age at 5 Years Visit (years) | 5.0 [5.0, 5.1] | 5.0 [5.0, 5.1] |
| Missing (n, %) | 1 (0.1) |  |
| Hours of Organized Physical Activity at 5 Years (per week) | 2.0 [1.0, 3.0] | 2.0 [1.0, 3.0] |
| Missing (n, %) | 304 (13.7) |  |
| Hours of TV Watching at 5 Years (per day) | 3.0 [2.0, 4.5] | 3.0 [2.0, 4.5] |
| Missing (n, %) | 276 (12.4) |  |

Values are frequency counts and percentages for categorical variables, means (SD) for continuous variables with a normal distribution, or medians (25th to 75th percentiles) for continuous variables with a skewed distribution.
BMI = Body mass index.

## **Table S3**. Descriptive characteristics of participants in the full cohort vs those included in the analysis: the CHILD Cohort Study

|  |  | **Children Included in the Analyses** |
| --- | --- | --- |
|  | **Full Cohort** |  |
|  | **(N = 3454)** | **(N = 2219)** |
| **Family Characteristics** |  |  |
| Maternal Age (years) | 32.3 (4.7) | 32.5 (4.5) |
| Missing (n, %) | 192 (5.6) | 0 (0.0) |
| Maternal BMI (kg/m^2^) | 23.3 [21.0, 27.1] | 23.1 [21.0, 26.7] |
| Missing (n, %) | 518 (15.0) | 67 (3.0) |
| Maternal Education (Post-secondary Education vs. None) | 2389 (69.2) | 1715 (77.3) |
| Missing (n, %) | 327 (9.5) | 52 (2.3) |
| Household Income |  |  |
| $0 - $99,999 | 1342 (38.8) | 914 (41.2) |
| > $100,000 | 1494 (43.3) | 1061 (47.8) |
| Prefer Not to Say | 294 (8.5) | 192 (8.7) |
| Missing (n, %) | 324 (9.4) | 52 (2.3) |
| Pregnancy Stress Score | 12.0 [8.0, 16.0] | 11.1 [8.0, 16.0] |
| Missing (n, %) | 469 (13.6) | 132 (5.9) |
| Study Site |  |  |
| Edmonton | 691 (20.0) | 492 (22.2) |
| Manitoba | 976 (28.3) | 705 (31.8) |
| Toronto | 726 (21.0) | 483 (21.7) |
| Vancouver | 704 (20.4) | 539 (24.3) |
| Missing (n, %) | 357 (10.3) | 0 (0.0) |
| **Birth Characteristics** |  |  |
| Child Sex (Males vs. Females) | 1816 (52.6) | 1175 (53.0) |
| Missing (n, %) | 0 (0.0) | 0 (0.0) |
| Birth Weight (grams) | 3444.3 (480.9) | 3457.9 (476.1) |
| Missing (n, %) | 86 (2.5) | 45 (2.0) |
| Gestational Age at Birth (weeks) | 39.7 [38.9, 40.4] | 39.7 [38.9, 40.6] |
| Missing (n, %) | 61 (1.8) | 32 (1.4) |
| Child Ethnicity |  |  |
| Caucasian White | 2046 (59.2) | 1447 (65.2) |
| Multiracial | 745 (21.6) | 512 (23.1) |
| Other | 420 (12.2) | 252 (11.3) |
| Missing (n, %) | 243 (7.0) | 8 (0.4) |
| Older Siblings (Yes vs. No) | 1452 (42.0) | 995 (44.8) |
| Missing (n, %) | 292 (8.5) | 28 (1.3) |
| Exclusive Breastfeeding at 3 Months (Yes vs. No) | 1884 (54.5) | 1371 (61.8) |
| Missing (n, %) | 303 (8.8) | 7 (0.3) |
| Breastfeeding Duration (months) | 10.0 [5.0, 14.0] | 11.0 [6.0, 15.0] |
| Missing (n, %) | 297 (8.6) | 5 (0.2) |
| **Childhood Characteristics** |  |  |
| Age at 3 Years Visit (years) | 3.0 [3.0, 3.1] | 3.0 [3.0, 3.1] |
| Missing (n, %) | 568 (16.4) | 45 (2.0) |
| Daily Caloric Intake at 3 Years (kcal/day) | 1400.4 [1142.3, 1718.7] | 1395.1 [1142.0, 1706.0] |
| Missing (n, %) | 1020 (29.5) | 0 (0.0) |
| Study Season at 3 Years Diet Assessment |  |  |
| Autumn | 578 (16.7) | 518 (23.3) |
| Spring | 657 (19.0) | 603 (27.2) |
| Summer | 621 (18.0) | 563 (25.4) |
| Winter | 577 (16.7) | 535 (24.1) |
| Missing (n, %) | 1021 (29.6) | 0 (0.0) |
| Age at 5 Years Visit (years) | 5.0 [5.0, 5.1] | 5.0 [5.0, 5.1] |
| Missing (n, %) | 658 (19.1) | 1 (0.1) |
| Hours of TV Watching at 5 Years (per day) | 3.0 [2.0, 5.0] | 3.0 [2.0, 4.5] |
| Missing (n, %) | 1177 (34.1) | 276 (12.4) |
| **Body Composition and Obesity** |  |  |
| BMI z-score (WHO) at 5 Years (kg/m^2^) | 0.3 (1.0) | 0.3 (1.0) |
| Missing (n, %) | 664 (19.2) | 3 (0.1) |
| Waist Circumference z-score (NHANES III) at 5 Years (cm) | 0.0 (1.0) | -0.1 (1.0) |
| Missing (n, %) | 836 (24.2) | 18 (0.8) |
| Obesity + Overweight at 5 Years (Yes vs. No) | 575 (16.6) | 444 (20.0) |
| Missing (n, %) | 664 (19.2) | 3 (0.1) |
| Obesity at 5 Years (Yes vs. No) | 142 (4.1) | 105 (4.7) |
| Missing (n, %) | 664 (19.2) | 3 (0.1) |

Values are frequency counts and percentages for categorical variables, means (SD) for continuous variables with a normal distribution, or medians (25th to 75th percentiles) for continuous variables with a skewed distribution. Values are based on the original data. BMI = Body mass index. WHO = World Health Organization; NHANES III = The Third National Health and Nutrition Examination Survey.

## **Figure S2.** Associations of dietary patterns at three years of age and body mass index and waist circumference z-scores at five years of age from the basic model, stratified by sex in the CHILD Cohort Study (N=2219)


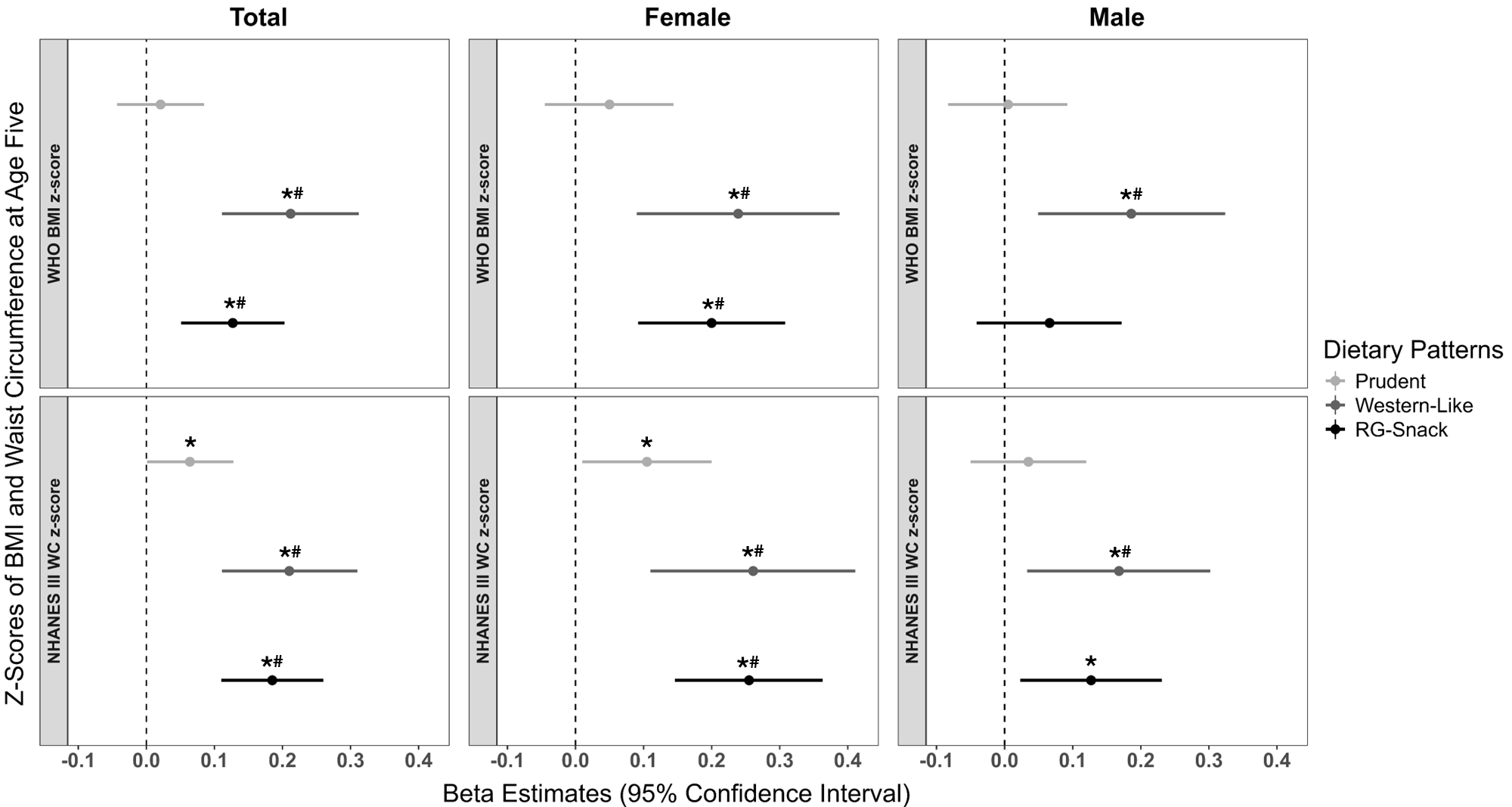


Values are beta estimates with 95% confidence intervals from linear regression models of dietary patterns at three years of age (Prudent, Western-Like and Refined Grain–Snack (RG-Snack)), and body mass index (BMI) and waist circumference (WC) z-scores at five years of age in the CHILD Cohort Study (N=2219), stratified for Female (N=1044) and Male (N=1175). The model was adjusted for total energy intake at age three. *p-value<0.05 and # significance remained after Bonferroni correction. WHO = World Health Organization; NHANES III = The Third National Health and Nutrition Examination Survey.

## **Figure S3**. Multivariable-adjusted associations of dietary patterns, body mass index and waist-circumference z-scores additionally accounting for one-year body mass index, stratified by sex in the CHILD Cohort Study (N=2162)


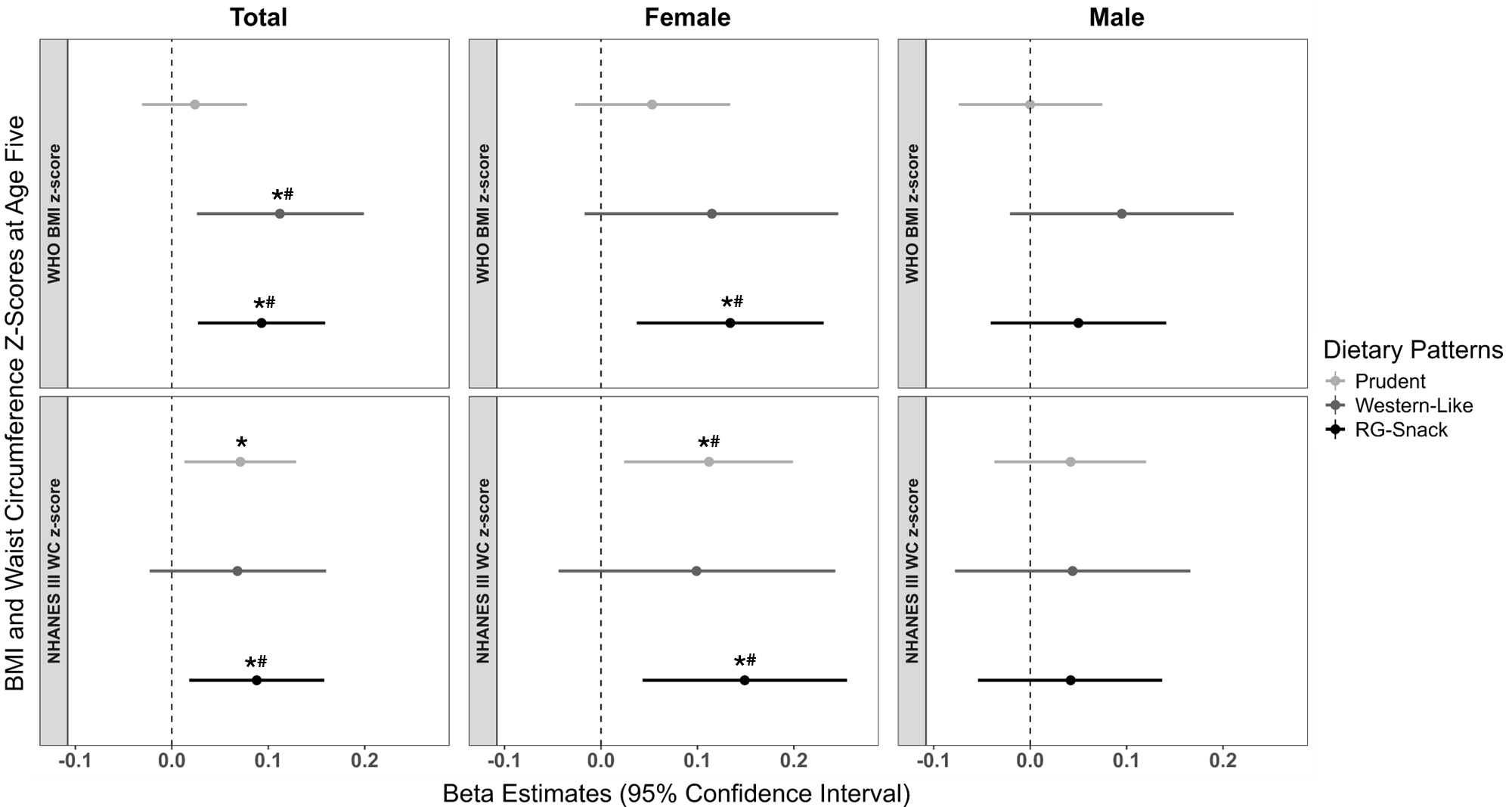


Values are beta estimates with 95% confidence intervals from linear regression analyses of dietary patterns at three years of age (Prudent, Western-Like and Refined Grain–Snack (RG-Snack)), and body mass index (BMI) and waist circumference (WC) z-scores at five years of age in the CHILD Cohort Study (N=2162), stratified for Female (N=1018) and Male (N=1144). The model was multivariable-adjusted for maternal age, pre-pregnancy BMI, postsecondary education, pregnancy stress, household income, study site, weight and gestational age at birth, child ethnicity, breastfeeding exclusivity and duration, having older siblings, screen time, total energy intake at age three, season of dietary assessments and BMI at one year of age. *p-value<0.05 and # significance remained after Bonferroni correction. WHO = World Health Organization; NHANES III = The Third National Health and Nutrition Examination Survey.

## **Figure S4**. Multivariable-adjusted associations of dietary patterns, body mass index and waist-circumference z-scores additionally accounting for three-year body mass index and change in energy intake, stratified by sex in the CHILD Cohort Study (N=1898)


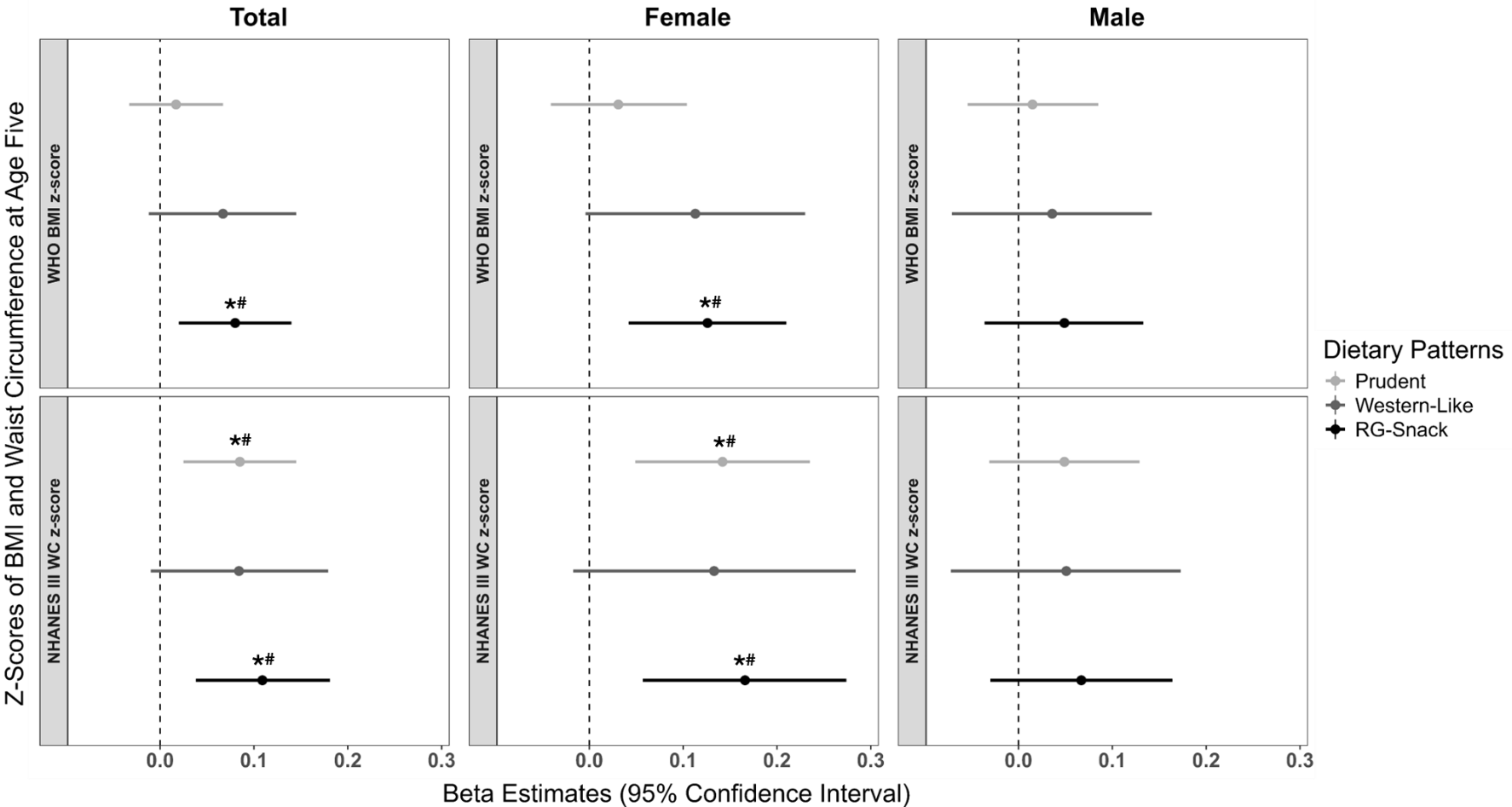


Values are beta estimates with 95% confidence intervals from linear regression analyses of dietary patterns at three years of age (Prudent, Western-Like and Refined Grain–Snack (RG-Snack)), and body mass index (BMI) and waist circumference (WC) z-scores at five years of age in the CHILD Cohort Study (N=1898), stratified for Female (N=884) and Male (N=1014). The model was multivariable-adjusted for maternal age, pre-pregnancy BMI, postsecondary education, pregnancy stress, household income, study site, weight and gestational age at birth, child ethnicity, breastfeeding exclusivity and duration, having older siblings, screen time, total energy intake at age three, season of dietary assessments, Δ energy intake between three and five and BMI at three year of age. *p-value<0.05 and # significance remained after Bonferroni correction. WHO = World Health Organization; NHANES III = The Third National Health and Nutrition Examination Survey.

## **Figure S5.** Multivariable-adjusted associations of dietary patterns at three years of age and obesity status (CDC Standards) at five years of age, stratified by sex in the CHILD Cohort Study (N=2217)


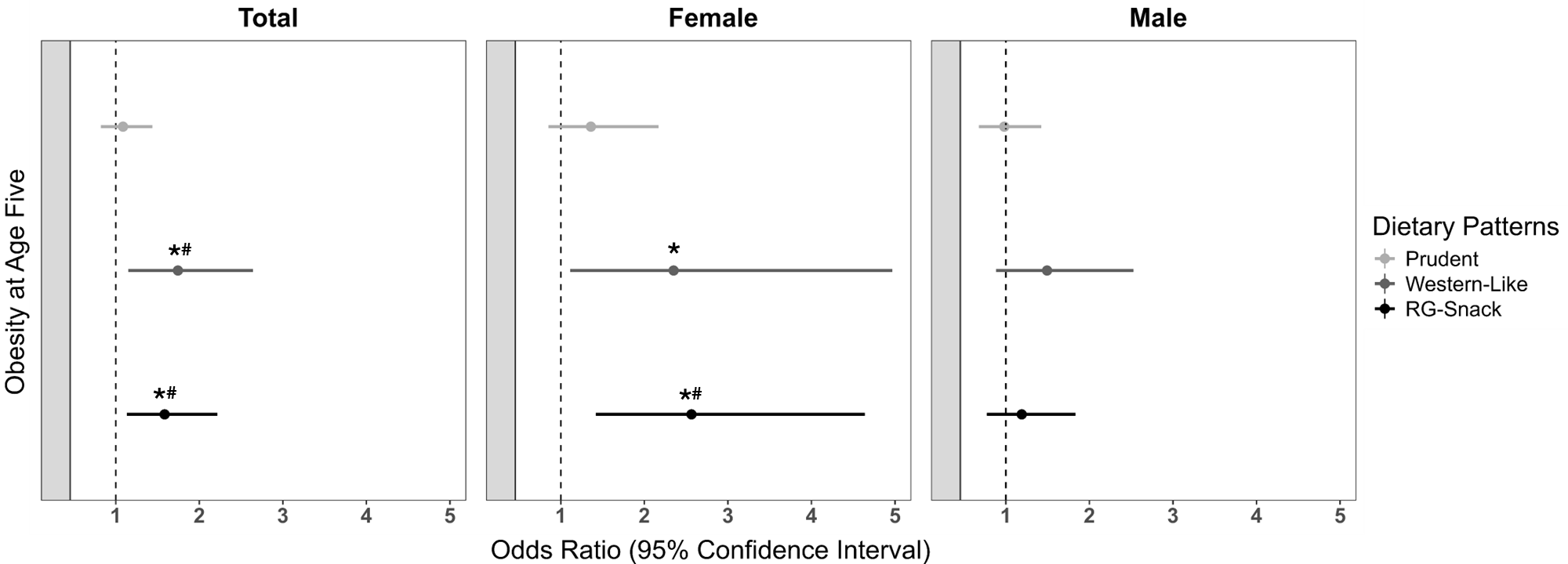


Values are odds ratios with 95% confidence intervals from logistic regression analyses of dietary patterns at three years of age (Prudent, Western-Like and Refined Grain–Snack (RG-Snack)) and obesity status at five years of age (Centers for Disease Control and Prevention body mass index (BMI) percentiles ≥ 95th) in the CHILD Cohort Study (N=2217), stratified for Female (N=1044) and Male (N=1173). The multivariable-adjusted model accounted for maternal age, pre-pregnancy BMI, postsecondary education, pregnancy stress, household income, study site, weight and gestational age at birth, child ethnicity, breastfeeding exclusivity and duration, having older siblings, screen time, total energy intake at age three and season of dietary assessments. *p-value<0.05 and # significance remained after Bonferroni correction.

## **Figure S6.** Multivariable-adjusted associations of dietary patterns at three years of age and overweight/obesity status at five years of age, stratified by sex in the CHILD Cohort Study (N=2216)


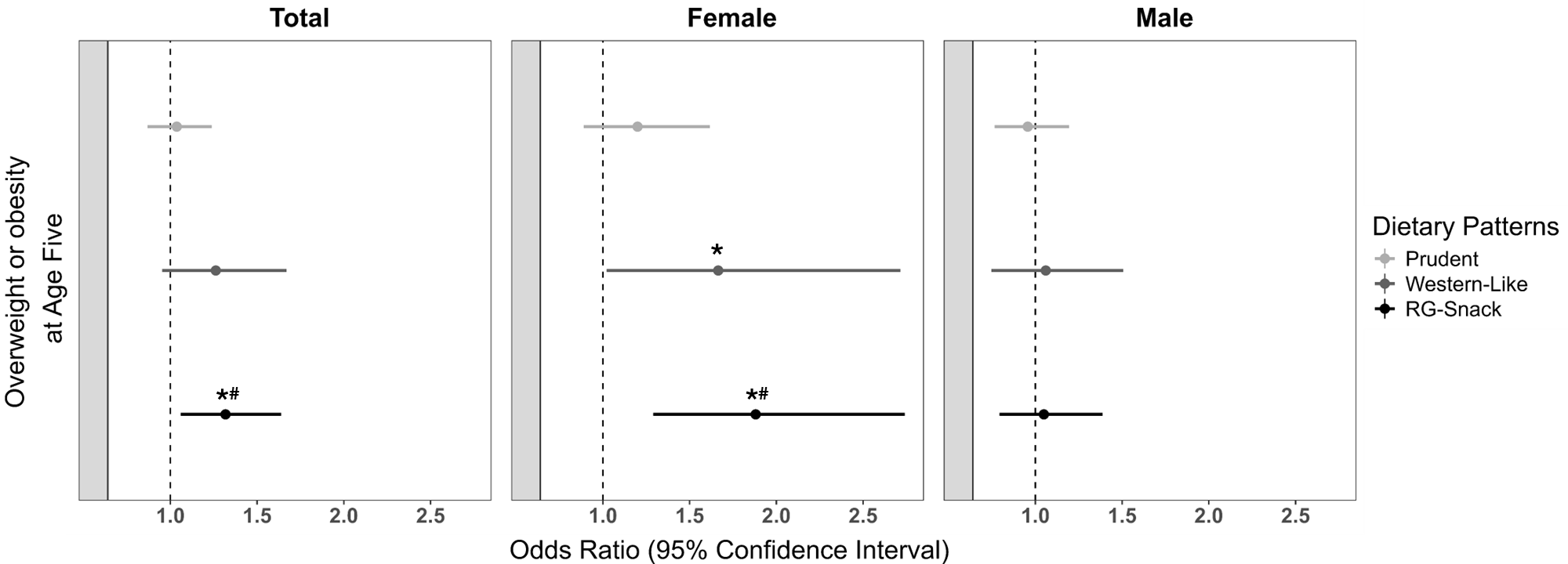


Values are odds ratios with 95% confidence intervals from logistic regression analyses of dietary patterns at three years of age (Prudent, Western-Like and Refined Grain–Snack (RG-Snack)) and overweight/obesity status at five years of age (World Health Organization body mass index (BMI) z-score > 1) in the CHILD Cohort Study (N=2216), stratified for Female (N=1044) and Male (N=1172). The multivariable-adjusted model accounted for maternal age, pre-pregnancy BMI, postsecondary education, pregnancy stress, household income, study site, weight and gestational age at birth, child ethnicity, breastfeeding exclusivity and duration, having older siblings, screen time, total energy intake at age three and season of dietary assessments. *p-value<0.05 and # significance remained after Bonferroni correction.
